# Evidence Factors in Fuzzy Regression Discontinuity Designs with Sequential Treatment Assignments

**DOI:** 10.1017/psy.2025.10033

**Published:** 2025-08-08

**Authors:** Youjin Lee, Youmi Suk

**Affiliations:** 1 Department of Biostatistics, Brown University, Providence, RI, USA; 2 Grace Dodge Hall 552, Teachers College, Columbia University, 525 West 120th Street, New York, NY 10027

**Keywords:** evidence factors (EFs), observational studies, regression discontinuity, sensitivity analysis

## Abstract

Many observational studies often involve multiple levels of treatment assignment. In particular, fuzzy regression discontinuity (RD) designs have sequential treatment assignment processes: first based on eligibility criteria, and second, on (non-)compliance rules. In such fuzzy RD designs, researchers typically use either an intent-to-treat approach or an instrumental variable-type approach, and each is subject to both overlapping and unique biases. This article proposes a new evidence factors (EFs) framework for fuzzy RD designs with sequential treatment assignments, which may be influenced by different levels of decision-makers. Each of the proposed EFs aims to test the same causal null hypothesis while potentially being subject to different types of biases. Our proposed framework utilizes the local RD randomization and randomization-based inference. We evaluate the effectiveness of our proposed framework through simulation studies and two real datasets on pre-kindergarten programs and testing accommodations.

## 1 Introduction

In observational studies, researchers often conduct multiple analyses on a single dataset to answer the same or related causal questions. This strategy can help rule out non-causal explanations and enhance the robustness of causal conclusions (Cook, 1985). However, if not carefully designed, multiple analyses may not be more beneficial than a single analysis; they can provide redundant information or consistently incorrect answers if they share overlapping biases. Planning and conducting a carefully designed multifaceted approach within a single dataset have motivated a new design known as *evidence factors* (EFs), introduced by Rosenbaum (2010). Briefly, EFs are two or more independent tests of the same causal null hypothesis about the treatment effect using a single dataset, with each potentially being subject to different biases. Each EF provides *unique* information about the causal question, and combining these factors can strengthen causal conclusions. The overarching goal of this article is to propose a new framework for constructing EFs in regression discontinuity (RD) designs.

An RD design, introduced by Thistlethwaite & Campbell (1960), is one of the most credible quasi-experimental designs. RD designs are used when a subject’s eligibility for treatment depends solely on a running variable (Hahn et al., 2001; Thistlethwaite & Campbell, 1960). If the value of the running variable (e.g., English proficiency) is below or equal to a cutoff, the subject is eligible for the treatment (e.g., a testing accommodation); otherwise, the subject is ineligible. This is known as a *sharp* RD design. However, eligibility does not necessarily lead to treatment use, and noncompliance yields a *fuzzy* RD design. For example, in a fuzzy RD setting, policy makers might establish the treatment eligibility rule, while the ultimate decision to use the treatment depends on the students themselves. As such, fuzzy RD designs inherently involve sequential assignment processes, where each is driven by different decision-makers. See Section 2 for two motivating examples from fuzzy RD designs.

When data are collected from fuzzy RD designs, researchers can use various analytic approaches to estimate causal effects. For example, one can apply an intent-to-treat approach (i.e., a sharp RD method) to estimate a causal effect at or near the cutoff, or one can use eligibility status as an instrumental variable (IV) to estimate a subgroup causal effect near the cutoff (Lee & Lemieux, 2010). However, both intent-to-treat and IV-based approaches in fuzzy RD designs focus on a local effect at or near the cutoff and are potentially vulnerable to the same bias associated with the eligibility rule, such as manipulation of the running variable, i.e., subjects precisely adjusting its values (Cattaneo et al., 2024; Crespo, 2020; Lee & Lemieux, 2010). Alternatively, researchers may consider an as-treated analysis that compares subjects who actually used the treatment with those who did not, to estimate the treatment effect. While this approach ensures greater generalizability, it is susceptible to confounding bias due to self-selection. Each analytic approach relies on different sets of causal assumptions and has unique strengths and weaknesses, so researchers may benefit from applying multiple approaches within a single dataset (Suk et al., 2022; Wong et al., 2007).

However, simply conducting multiple analyses does not lead to more accurate causal conclusions. If not properly designed, multiple analyses may only be slight variations subject to the same bias. In contrast, EF analysis aims to carefully design (nearly) independent analyses and then combine them to reinforce causal conclusions on the same null hypothesis. Each EF is designed to provide a unique and independent piece of evidence on the treatment effect. Recent studies have extended the original idea of Rosenbaum (2010) to different settings (Karmakar & Small, 2023; Rosenbaum, 2023; Zhao et al., 2022). For example, Karmakar & Small (2023) and Zhao et al. (2022) propose EF analysis with IVs, and Rosenbaum (2023) introduces EFs using a second control (see Section 3.2 for more details). Although these prior studies can touch on certain features of fuzzy RD designs through IV-type analyses, none explicitly tackle unique features and challenges inherent in RD settings. Fuzzy RD designs offer a distinct setup due to their inherent locality and sequential assignment processes, neither of which has been addressed in EFs analysis.

In this article, we propose a novel framework for constructing EFs in fuzzy RD designs with sequential treatment assignments. We achieve this by restricting the sample within the window for one analysis and properly conditioning on past treatment assignments in the subsequent analysis. Our proposed framework involves three key steps: (i) constructing EFs, (ii) combining *p*-values from each EF, and (iii) conducting sensitivity analysis. With the proposed framework, we contribute to both RD and EFs literature as follows. First, we introduce the EFs design into fuzzy RD analysis by leveraging the inherent causal relationships among treatment variables to disentangle biases across multiple analyses. Unlike traditional fuzzy RD approaches, which rely on intent-to-treat and IV-type analyses that suffer from the overlapping biases, our EF framework provides nearly independent pieces of evidence from multiple analyses, both statistically and causally, thereby strengthening causal conclusions. Second, we formalize the causal assumptions required for constructing multiple EFs in fuzzy RD designs, a new setting for EF analysis. Specifically, we leverage sequential decision-making processes and the locality inherent in analyzing samples near the cutoff to construct valid EFs. Importantly, our design accommodates both nested and non-nested structures among treatment assignment variables and uses a single dataset from a fuzzy RD design without requiring external data. Lastly, we extend sensitivity analysis methods used in EFs designs to the fuzzy RD setting. In particular, we incorporate an assignment model and test statistic that reflect locality in RD designs, and use them in sensitivity analyses to assess the robustness of our conclusions to violations of the key assumption of the RD design.

The remainder of this article is structured as follows. Section 2 illustrates two real-world, fuzzy RD examples, exhibiting one-sided and two-sided non-compliance, respectively. Section 3 provides a brief literature review on RD and EFs. Section 4 introduces our settings and formalizes our assignment models with their causal assumptions. Section 5 then describes our proposed EF approach and its properties. Section 6 provides simulation studies, and Section 7 presents two data applications. Lastly, we provide our discussion and conclusions in Section 8.

## 2 Two motivating examples

### 2.1 State pre-kindergarten (pre-K) program

**Figure 1:**
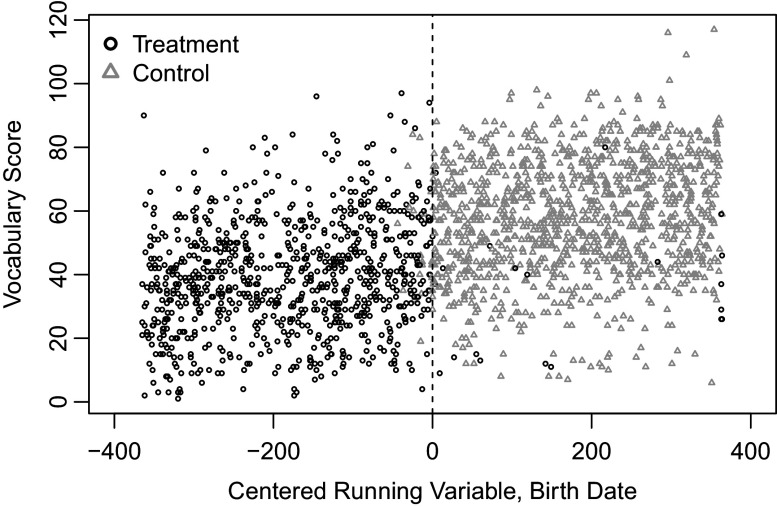
Visual representation of a fuzzy RD design in New Jersey’s pre-K program: black points represent the treatment group (i.e., program user group), while gray points represent the control groups (i.e., non-user group).

Wong et al. (2007) investigate whether state pre-K programs for 4-year-old children improve academic performance in their vocabulary, math, and print awareness skills. This study employs an RD design where the running variable is a child’s birth date; children with birthdays on or after a certain date are eligible for enrolling in the pre-K program, whereas those with birthdays before it are not. Additionally, there are non-compliers who do not follow the treatment assignment based on eligibility, and the presence of these non-compliers requires using a fuzzy RD design for program evaluation (Suk, 2024). Figure 1 provides an RD plot showing the relationship between the outcome of vocabulary scores and the running variable of birth date. While the majority of children comply with the eligibility status determined by the running variable, we observe non-compliers on both sides of the cutoff, i.e., two-sided non-compliance.

Rosenbaum (2023) introduces how to construct EFs with multiple control groups to test a single hypothesis, where each factor is vulnerable to different biases. To evaluate the effect of New Jersey’s pre-K program, we can adapt this approach by conducting an EF analysis to test the same null hypothesis: there is no effect of using the pre-K program. However, this application raises some important questions. First, is Rosenbaum (2023)’s proposal directly applicable to a fuzzy RD design with two-sided non-compliance? Second, how can we form multiple comparisons for EFs analysis in the fuzzy RD design? Third, how many valid EFs can be constructed in such a design?

### 2.2 Extended time accommodation (ETA)

Testing accommodations are essential for students with disabilities or English language learners (ELLs) to accurately demonstrate their abilities during assessments. The ETA is the most frequently provided testing accommodation in many testing programs. Suk et al. (2022) and Suk & Kim (2024) examine the effects of ETA for ELLs using a fuzzy RD design. They use ELL English proficiency scores as the running variable, and ETA eligibility status as an IV. In ETA settings, sequential assignment processes with one-sided non-compliance occur, and they result in three treatment statuses: eligibility, receipt, and use; see Table 1 for treatment statuses and four different groups formed through the sequential assignment process.

**Table 1:** Treatment statuses and four different groups in the context of the ETA

ETA eligibility status	ETA receipt status	ETA used status
0	0	0
1	0	0
1	1	0
1	1	1

Prior studies (Suk & Kim, 2024; Suk et al., 2022) assume that the ETA effect is only present when students make use of it; otherwise, there is no assumed ETA effect. They employ a fuzzy RD design to estimate three causal effects: the effect of being eligible for ETA at the cutoff score, the effect of receiving ETA at the cutoff among compiler students, and the effect of using ETA at the cutoff among compiler students.^1^ While these different causal effects provide informative evidence on ETA’s effectiveness, each effect estimate could be susceptible to different types of biases occurring at each assignment process, as well as to the overlapping biases inherent in the RD design (e.g., manipulation of the running variable). However, if carefully designed, EFs can be constructed in this ETA data to test the same null hypothesis of “no effect of using ETA,” where each factor has independent pieces of information about the null hypothesis.

## 3 Literature review and our contribution

### 3.1 Local randomization framework for RD designs

Our setting is based on RD designs, and there are two frameworks available: continuity-based and local randomization. The first framework is based on the continuity assumption (Hahn et al., 2001). This framework assumes that the conditional expectations of potential outcomes, given the running variable, are continuous at the cutoff to provide valid counterfactuals in both sharp RD and fuzzy RD designs (Cattaneo et al., 2023b; Dong, 2018). This assumption often requires (local) parametric or nonparametric regressions. The second framework is the local randomization framework, which was motivated by the works of Lee (2008) and Thistlethwaite & Campbell (1960). This framework interprets RD designs heuristically as locally randomized experiments within a neighborhood of the cutoff (i.e., within the window, denoted by 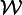 ). It is based on a local unconfoundedness assumption, which assumes that the treatment assignment (e.g., eligibility) is unconfounded given the observed covariates within the window 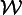 (Cattaneo et al., 2015). The main distinction between continuity-based and local randomization frameworks lies in how they formalize the idea of comparability (Cattaneo et al., 2024). While comparability under the continuity-based framework arises from extrapolating the limiting distribution of local regression-based estimators at the cutoff, the local randomization framework assumes comparability within a small neighborhood around the cutoff, like in randomized experiments, and utilizes randomization-based inference without having to specify regression models.

In this work, we adopt the second framework of the local randomization based on randomization-based inference, which utilizes the randomization mechanisms of treatments while treating the observed outcomes as fixed. Randomization-based inference not only accommodates the local randomization framework but also easily accounts for other commonly used designs for causal comparisons, such as matching and stratification (Rosenbaum, 2002). Randomization-based inference, which does not rely on large sample approximations, is particularly useful in fuzzy RD designs where at least one assignment depends on observations within a small window around the cutoff. When leveraging randomization-based inference, we assume that matched data on observed covariates come from a randomized trial; in other words, within the matched strata, the treatment status is randomly assigned to each subject. Therefore, an outcome comparison within the matched strata can provide a valid causal comparison, without additional covariate adjustment. Under the local randomization framework, such unconfoundedness must hold within one stratum that includes only subjects within a window around the cutoff. However, further covariate adjustment (e.g., matching) can be applied within this stratum to improve efficiency and power in estimating causal effects (Cattaneo et al., 2023a).

### 3.2 EF design with multiple analyses

An EF design can be used when multiple analyses are possible to test the same causal null hypothesis in observational studies. Instead of treating one among several analyses as primary and the others as secondary, each analysis contributes an independent piece of evidence in the EF design. Multiple analyses can arise from multiple doses (variations) of the treatment. For example, one analysis compares outcomes between the treated and control groups (e.g., smokers and non-smokers), while another analysis compares outcomes between those with intense treatment and those with weak treatment (e.g., heavy smokers and light smokers) within the treated group (Rosenbaum, 2010). To ensure these multiple analyses produce independent inferential results, Rosenbaum (2010) proposes an EFs design with multiple doses of the treatment.

Additionally, multiple control groups, who did not take the treatment for different reasons, can generate multiple analyses, where each control group is compared to the corresponding treatment group (Rosenbaum, 2023). Most recently, Rosenbaum (2023) introduces an EF design with multiple control groups. Specifically, Rosenbaum (2023) introduces two assignment variables, one of which defines secondary control, likely derived from external data sources, and deliberately nests these variables by design, even though their causal relationships are unclear. In contrast, as will be elaborated in the next section, our proposed EF design explicitly assumes causal relationships among treatment assignment variables within a single dataset. This assumption is valid because fuzzy RD designs inherently involve sequential assignment processes, influenced by different decision-makers. Our approach leverages these sequential assignment variables for EF analysis and accommodates both one-sided (nested) and two-sided (non-nested) non-compliance, each of which is illustrated in Section 2. This differs from the nested structure addressed in Rosenbaum (2023).

Furthermore, the presence of multiple IVs generates multiple analyses. These IV analyses are often correlated with each other as well as with a direct comparison between the treated and control groups. Recent research has explored EFs using such IVs (Karmakar & Small, 2023; Karmakar et al., 2020; Zhao et al., 2022). In particular, Karmakar et al. (2020) propose the reinforced design, where multiple IVs, in addition to a direct comparison (e.g., between actual users and non-users), provide EFs. Due to potential correlation among IVs, the reinforced design requires specifying the order of multiple analyses to avoid overlapping biases. Zhao et al. (2022) relax this ordering requirement by blocking or nesting procedures, each of which would only be valid under particular structures among IVs. In our context, multiple treatment assignments up to the final one can be considered IVs; however, these assignments must be causally ordered. This new requirement has not been formally addressed in the existing EF literature.

## 4 Settings

### 4.1 Notation

Subjects are grouped into strata using observed covariates to form *J* strata. We assume that the observed covariates of subject *i* in stratum *j* (subject 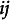 afterward), denoted by 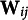 , are controlled by this stratification. In other words, 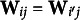 for all 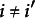 across all strata 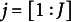 , where 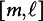 denotes a set of an integer from *m* to 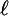 . Let 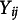 denote the outcome of interest for unit 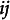 . Consider that the first level of treatment assignment exhibits an RD design, determining eligibility for treatment. Let 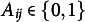 denote the eligibility for the treatment, where 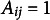 indicates that subject 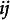 is eligible and not eligible if 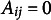 . The eligibility is determined based on a running variable, denoted by 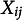 . Given a cutoff 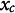 , 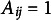 if 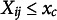 and 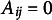 if 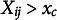 .^2^ Let 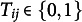 indicate the actual treatment use of subject 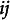 . However, not all eligible subjects actually use the treatment, and ineligible subjects occasionally use the treatment in practice. There could be sequential treatment assignment processes driven by different levels of decision-makers. For example, in the ETA application, (1) first, students’ eligibility is determined by their ELL English-proficiency scores according to government or state policy; (2) second, students receive ETA from their school administrators; and (3) lastly, students can choose to use the ETA offered.

Suppose that *K* denotes the number of treatment assignment processes ( 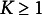 ), resulting in a *K*-dimensional treatment status for each subject. When 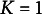 , we have the sharp RD with 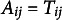 . When 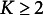 , 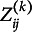 indicates that subject 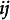 takes the treatment *at level k* ( 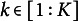 ) where 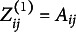 and 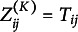 . We allow 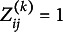 even if 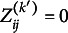 for any 
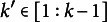
 , i.e., allowing two-sided non-compliance; for example, subject 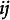 potentially uses the treatment even if they were ineligible and/or did not receive the treatment from school administrators. However, we focus on the assignment model with 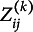 conditioning on 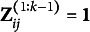 for each 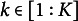 , where 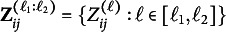 . For notational convenience, define 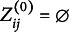 and 
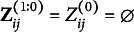
 . We elaborate this conditioning in Section 4.3. We denote 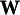 , 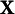 , 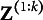 , and 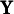 as the collection of 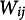 , 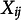 , 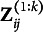 , and 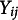 across units, respectively.

### 4.2 Potential outcomes, assumptions, and hypotheses

Let 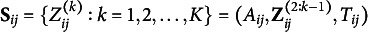 , which can take 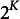 different values. Let 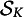 be the set of the 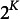 , length-*K* vectors with 0 or 1 coordinates. Under a series of one-sided non-compliance, a vector of 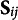 can take up to 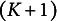 different values, where 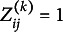 implies 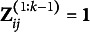 for all 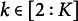 . For example, when 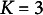 , 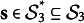 such that 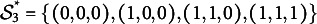 . Using the potential outcomes framework, let 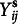 denote the potential outcome of 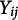 when 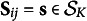 with 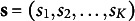 under the consistency assumption (Rubin, 1974). For example, 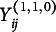 is the potential outcome of subject 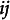 if she were eligible for the treatment and received the treatment from school administrators but did not use it. The following assumption is essential for using 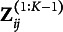 as a (conditional) IV to examine the causal effect of the actual treatment use.

Assumption 1.

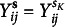
 for all 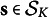

In other words, a whole vector of 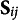 has an effect on the outcome only through the actual treatment use, 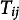 . This means the exclusion restriction (Angrist et al., 1996). Then, our null hypothesis of interest is as follows: (1)

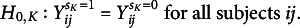

If our null of no treatment effect 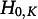 is true, then 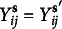 for all 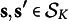 under Assumption 1. The null in (1) also implies that there is only one version of the potential control outcome among 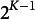 values in 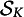 . Additionally, we require the following assumption about the (causal) relationship among *K* different treatment statuses, which represents the most distinct causal structure compared to the existing EF literature (Karmakar et al., 2020; Rosenbaum, 2023; Zhao et al., 2022).

Assumption 2. (a) Treatment statuses, 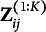 , are causally ordered and (b) there are no unknown or unmeasured common causes among them.

The condition (a) of Assumption 2 implies that the treatment status at level *k* is established *after* the treatment statuses at or earlier than level 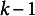 are determined. For example, students’ decision to use the treatment should not affect their eligibility and receipt statuses. Moreover, there should be no unmeasured confounding among the treatment statuses at different levels as implied in the second condition. This assumption is more likely to hold when distinct decision-makers determine the treatment assignment process at each level, each affected by different criteria or rules. For instance, in ETA settings, assignment decisions are made separately by entities, such as government or state policy, schools, and students. Each decision-maker is likely to rely on different information (e.g., state-level cutoffs, school-level logistics, and student preferences) while also considering some shared, observable information (e.g., students’ ELL English proficiency scores).

**Figure 2:**
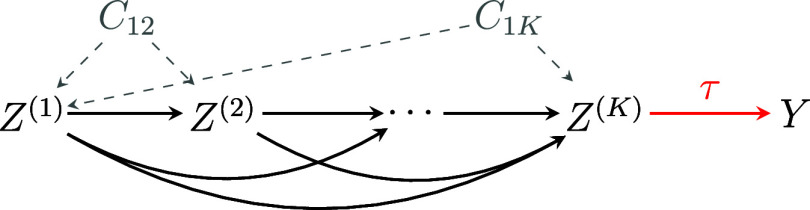
A directed acyclic graph (DAG) illustrating the causal relationships among treatment statuses 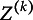 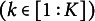 , outcome *Y*, and unmeasured common causes 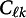 between 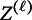 and 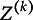 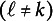 . *Note*: Observed covariates are omitted for simplicity.

To illustrate, Figure 2 provides a causal structure among variables under Assumption 1 and condition (a) of Assumption 2. In Figure 2, the treatment statuses of 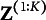 are causally ordered and any status among 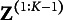 does not have a direct effect on the outcome *Y*. Condition (b) of Assumption 2 further assumes that there are no unmeasured common causes among 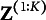 , such as 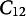 and 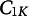 , that can confound the causal relationships among the treatment statuses.

We note that we can relax condition (b) of Assumption 2 to allow for unmeasured common causes among *some* of the *K* treatment statuses. In Section S2 of the Supplementary Material, we provide a method for selecting a valid set of treatment statuses by excluding one of the two statuses that are presumed to share unmeasured common causes.

### 4.3 Treatment assignment models

In this section, we illustrate the treatment assignment models at each level of a sequential assignment process, which can seamlessly incorporate Rosenbaum’s sensitivity analysis (Karmakar et al., 2020; Rosenbaum, 1987; Zhao et al., 2022). Let 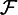 denote the collection of the potential outcomes and observed and unobserved covariates. Then, we posit the following assignment model at level 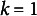 : (2)

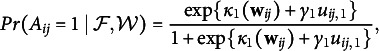

where 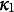 is an arbitrary function of the observed covariates, and 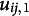 denotes the unmeasured covariate. When 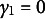 , this model entails the key assumption of the local randomization framework in an RD design, where eligibility is randomly assigned among subjects given the observed covariates within the window 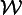 . In this case, 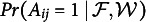 only depends on the observed covariates’ values, and thus, subjects within stratum *j* have the same probability of 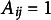 . Therefore, 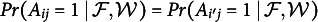 for all 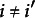 .

At subsequent levels of 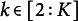 , we consider the following assignment model among those who were assigned to treatment at previous levels 
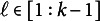
 : (3)

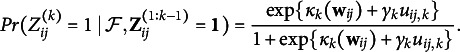

Similarly, 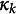 is an arbitrary function of the observed covariates, and 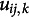 is the unmeasured covariate for each 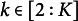 . Note that as stated in Assumption 2, unmeasured covariates at each assignment level are unique to that level and do not influence other levels. The model (3) implies that given that subject 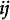 was assigned to treatment at previous levels 
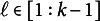
 , whether subject 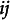 is assigned to treatment or not at level *k* depends on 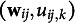 ; if 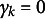 , then the assignment does not depend on any unobserved covariates.

We do not posit any model for treatment status 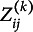 among subjects with 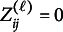 for *any* 
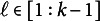
 and 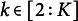 . In practice, it is expected that the assignment mechanisms among those subjects (e.g., assignment of receipt status among ineligible students) would be very different from those who were assigned to treatment at previous assignment processes. We consider the former case as “exceptions” or “nuisances,” while the latter assignment models are assumed to be known given 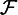 . In Section S3 of the Supplementary Material, we discuss alternative treatment assignment models for 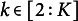 that further condition on the window 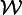 or the running variable 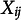 .

## 5 EFs in fuzzy RD designs

### 5.1 Constructing multiple comparisons

We propose constructing up to *K* number of test statistics (EFs) using 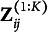 . We consider model-free (one-sided) test statistics, such as the stratified Wilcoxon’s signed rank statistic (Wilcoxon, 1992). We denote the test statistic at level *k* as 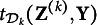 , where 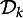 represents the subset of units used in the test, which possibly depends on 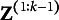 , 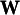 , and 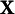 . The statistic 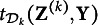 uses 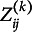 as the treatment assignment variable and 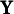 as fixed outcomes. Under Assumption 1 and the null hypothesis in (1), we have 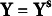 for all 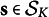 . Therefore, each statistic 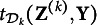 for 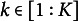 tests whether the potential outcomes under a particular treatment vector in 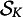 differ and can also provide a valid test for the null in (1) albeit with different power rates. For each test at level *k*, we refer to the group with 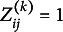 as the treatment comparison group, and the other group with 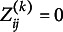 as the control comparison group.

Specifically, we first propose testing the null (1) with the test statistic 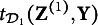 , using 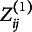 (or equivalently, 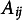 ) as a treatment assignment variable. We perform the RD analysis under the local randomization framework, resulting in the comparison only within the window 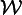 , i.e., 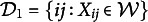 . This analysis would be valid to test for the null 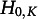 under Assumption 1 and would not be affected by any of 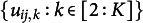 under Assumption 2. The subsequent EFs at level *k* ( 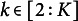 ) are constructed using only the subset of subjects with 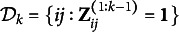 . By conditioning on 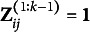 , any bias occurring at previous levels would not affect the validity of the test at level *k*.

Consider the simplest case with 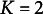 under two-sided non-compliance, as in our pre-K program example. Table 2 illustrates the treatment and control groups used for EF analysis within stratum *j*, stratified by the indicator for the window inclusion (i.e., whether 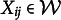 ) and their treatment statuses 
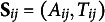
 . Two levels of treatment assignments result in two control groups: the first control group (Control 1) consists of subjects assigned control at 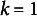 , while the second control group (Control 2) consists of subjects assigned treatment at 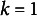 but control at 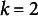 . The treated group contains the actual users of the treatment. In this case, our proposed framework allows us to construct two EFs. The first EF analysis (EF 1 in Table 2) uses only subjects within the window and compares ineligible subjects with 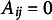 to eligible subjects with 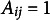 . Based on a value of 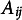 , this analysis pretends that the subjects in the second control group are part of the treatment comparison group. It compares the first control group to the other two groups within a window 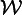 (i.e., those with 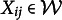 ). This analysis corresponds to the intent-to-treat analysis in an RD design, which only uses the eligibility assignment, but ignores the actual treatment use. On the other hand, the second EF analysis (EF 2 in Table 2) only compares the second control group and the treated group while excluding the first control group (e.g., any ineligible subjects) from the analysis but includes those outside of the window.

**Table 2:** Treatment statuses and the treatment and control comparison groups used for each EF analysis, resulting from a two-level treatment assignment process ( 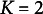 ) with two-sided non-compliance

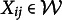	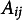 (Treatment eligible)	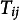 (Treatment used)	Group	EF 1	EF 2
1	0	0	Control 1	C	
1	0	1	Control 1	C	
1	1	0	Control 2	T	C
1	1	1	Treatment	T	T
0	0	0	Control 1		
0	0	1	Control 1		
0	1	0	Control 2		C
0	1	1	Treatment		T

*Note*: At each level, subjects with status 0 represent the control comparison group, while those with status 1 represent the treatment comparison group. T: Treatment comparison group; C: Control comparison group;  : Data excluded.

For further illustration, consider the case with 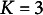 under one-sided non-compliance as in the ETA study. In the ETA setting, we have three different control groups: the first control group consists of subjects with 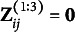 ; the second control group consists of subjects with 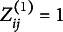 but with 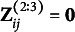 ; and the third control group includes those with 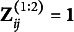 but with 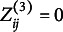 (see Table 3). With our proposed framework, we can construct three EFs. The first EF includes all of these three control groups using 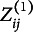 as the treatment assignment variable within a window 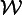 ; the second EF excludes the first control group and uses 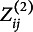 as the assignment variable; and lastly, the third EF only compares the last control group to the treated group. In both Tables 2 and 3, we do not use variation in the treatment status 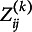 among those with 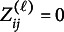 for any 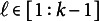 .

**Table 3:** Treatment statuses and the treatment and control comparison groups used for each EF analysis, resulting from a three-level treatment assignment process ( 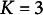 ) with one-sided non-compliance

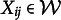	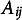 (Treatment eligible)	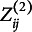 (Treatment received)	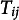 (Treatment used)	Group	EF 1	EF 2	EF 3
1	0	0	0	Control 1	C		
1	1	0	0	Control 2	T	C	
1	1	1	0	Control 3	T	T	C
1	1	1	1	Treatment	T	T	T
0	0	0	0	Control 1			
0	1	0	0	Control 2		C	
0	1	1	0	Control 3		T	C
0	1	1	1	Treatment		T	T

*Note*: At each level, subjects with status 0 represent the control comparison group, while those with status 1 represent the treatment comparison group. T: Treatment comparison group; C: Control comparison group;  : Data excluded.

### 5.2 Randomization-based inference and multiple *p*-values

We apply randomization-based inference for testing the treatment effect with multiple comparisons at each level 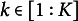 . With the test statistic 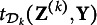 for 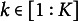 , we assume that all outcomes 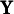 are fixed and randomization of treatment assignment 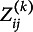 is the only source of randomness (Rosenbaum, 2002). The assignment models provided in (2) and (3) enable us to leverage randomization mechanisms (within strata) in a finite population, avoiding any specific modeling or reliance on asymptotic conditions. Without an unmeasured covariate in each treatment assignment model (i.e., 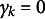 for 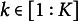 ), the treatment status 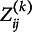 is randomly assigned to each subject within each stratum, which is constructed from the observed covariates within the window 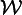 if 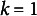 , or conditional on 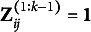 if 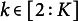 .

As a result of performing randomization-based inference at each level, we obtain one *p*-value for each comparison. Let 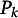 denote a *p*-value from the EF analysis at level *k* ( 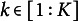 ). Suppose that there is no unmeasured covariate in each level *k*’s assignment model (i.e., when 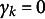 ). Then, under Assumption 1, we have 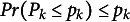 for all 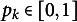 when the null 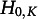 is true (i.e., each 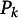 is a valid *p*-value for testing 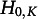 ). Theorem 1 further demonstrates the properties of the joint distribution among 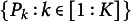 under the null 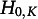 , where we use a nested conditioning structure in our test statistics 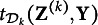 , for both one-sided and two-sided non-compliance among treatment assignment variables. See a detailed proof in Section S1 of the Supplementary Material.

Theorem 1. Under Assumption 1, suppose that the assignment models (2) and (3) hold with 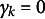 for all 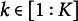 . Then *p*-values from the above design are stochastically larger than the uniform under the null. In other words, (4)

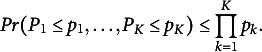

However, it is possible that some comparisons/analyses out of *K* assignments are invalid, i.e., biased. Let a subset 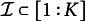 denote the analyses that are biased, while 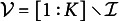 represents the set of indices that produce valid comparisons. For example, suppose that 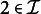 with 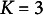 , where the second comparison is invalid due to an unmeasured covariate (i.e., 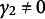 in model (3)). Then, the following results imply that this unmeasured covariate would not affect the properties of *p*-value in (4) among valid comparisons, e.g., 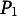 and 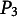 .

Theorem 2. Under Assumptions 1 and 2, (5)

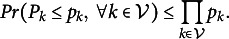

**Figure 3:**
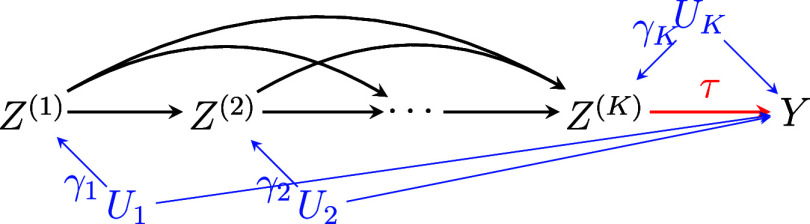
A DAG illustrating the hypothetical causal relationships among variables with unmeasured covariates, 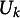 ’s, denoted in blue. *Note*: An edge between 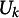 and 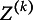 is present if and only if 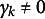 ( 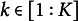 ). Observed covariates 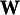 and running variable *X* are omitted for simplicity.

Theorem 2 demonstrates that the bias in the treatment assignments from other analyses would not affect the (near) independence properties among valid EFs, in addition to their individual validity; see a proof in Section S1 of the Supplementary Material. This independence is attributed to the fact that a value of 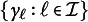 would not affect the validity of 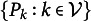 (see Figure 3).

In Figure 3, a variable 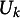 denotes an unmeasured covariate that can confound the relationship between 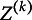 and 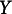 ( 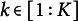 ) when 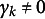 ( 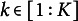 ). Then we have 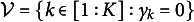 and 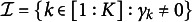 . Suppose that 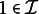 . Then bias due to 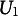 could represent bias in the local randomization in RD designs. This bias would not necessarily invalidate analyses at any level 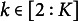 , as their analyses all condition on 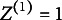 (e.g., conditioning on eligible subjects). Similar arguments can be made for the impact of 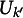 on analyses at level 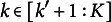 for each 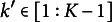 . Furthermore, the presence of 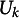 ( 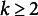 ) results in invalid analysis with 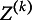 testing the null, but bias does not necessarily imply bias in 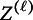 with 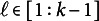 under the condition (b) of Assumption 2 that there is no unmeasured common cause between 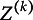 and 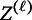 for 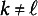 . For example, when 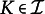 and 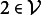 , bias due to 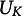 would not affect the validity of 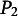 . This is because the condition (b) of Assumption 2 excludes the presence of unmeasured confounding between 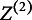 and *Y* through 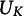 .

Based on Theorem 2, we can ensure that at least 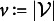 number of nearly independent *p*-values are obtained. Having nearly independent *p*-values enables us to easily isolate evidence from any set of *p*-values. In such a case, we can utilize *v* largest *p*-values among *K* to form a single valid *p*-value without specifying which *v* analyses provide valid EFs. Define 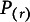 as the 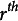 order statistic (i.e., 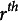 smallest value) of 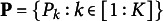 and let 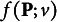 be the combining function of 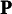 , with 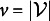 . Then with 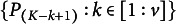 given *v*, the results from EFs can be combined into one valid *p*-value, using relatively simple methods of combining independent *p*-values, such as Fisher’s method or truncated product method (Zaykin et al., 2002), as follows: 

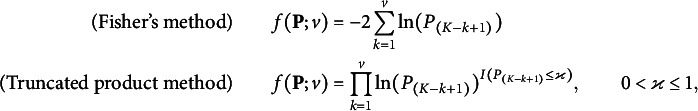

where 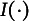 is an indicator function. The combined *p*-value is obtained using the known null distribution of each statistic 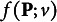 when at least *v* *p*-values in 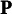 are (nearly) independent. This combined *p*-value from multiple EFs, compared to using individual *p*-values, can provide stronger evidence of the treatment effect when each factor suggests concurrent agreement about the null hypothesis.

We remark a potential concern regarding a lack of power, despite the validity of the test statistics 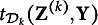 for 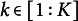 in testing the target null of 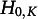 . This is because the treatment comparison group at each level *k* can include a large portion of subjects who do not actually use the treatment, potentially diluting the effect of the treatment at *k* level on the outcome. To avoid such dilution, researchers can use robust test statistics (see Section 4 of Rosenbaum (2023) for more details).

### 5.3 Sensitivity analysis

Researchers can use their conjecture on 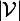 , say *q*, as a sensitivity parameter and examine the changes in their causal conclusions as a value of *q* varies ( 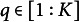 ). Researchers may reflect their prior knowledge about treatment assignment variables in their choice of *q*, for example, the maximum number of treatment assignments that would satisfy Assumption 2. Selecting *q* can easily lead to conservative results, however, as it discards 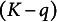 smallest *p*-values, which may contain *p*-values from valid EFs. Instead of discarding several small *p*-values, one can conduct sensitivity analysis to a specific unmeasured covariate in each assignment model by varying a value of parameter 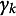 ’s in (2) and (3). A non-zero value of 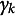 in (2) or (3) (or equivalently, strictly larger than one of 
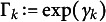
 ) implies a biased assignment at level *k* within strata formed by observed covariates ( 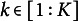 ).

Given 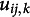 and 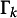 , assuming that the observed difference between the two comparison groups could be caused by this 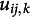 by an amount of 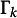 , one could calculate a corresponding *p*-value for testing the sharp null (1). This resulting *p*-value is often more conservative compared to the case with 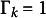 where all the observed differences are attributable to the existing causal effect. Since 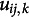 ’s are unknown, we can use the maximum *p*-value over 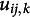 values in [0,1] when the sensitivity parameter is at most 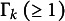 . We denote this adjusted *p*-value as 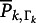 ( 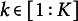 ). Then each of 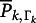 is a valid *p*-value and they also maintain the nearly independence properties under the null. This is because non-zero values of 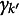 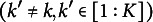 would not necessarily affect the validity of 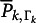 ; in other words, we have 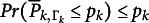 for any 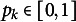 under the null regardless of the values of 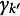 .

Similar to the argument regarding *q*, as a value of 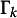 increases, we are more likely to obtain conservative inferences. This is because we consider the observed difference between two comparison groups to be partially attributable to existing imbalances between these groups on an unmeasured covariate. In such a sensitivity analysis, researchers may decide the presumed maximum level of 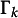 for each assignment *k* (e.g., eligibility assignment would be at most biased by 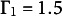 , while the assignment of treatment use would be at most biased by 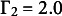 for those who are eligible), and then examine whether the results from multiple EFs are still against (or supporting) the null hypothesis.

## 6 Simulation study

We numerically evaluate the validity and the performance of our proposed approach through simulation studies that mimic the real data settings on pre-K programs and ETAs. We focus on examining (i) the non-overlapping bias between the proposed EFs and (ii) the validity of the combined *p*-value in the presence of invalid analyses. To examine (i), we intentionally introduce multiple sources of bias, each of which can invalidate at least one EF, and investigate whether one source can bias multiple factors. To examine (ii), we set 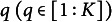 as the conjectured minimum number of valid analyses among *K*. Our goal is to demonstrate that the combined *p*-value given *q* using Fisher’s method, denoted as 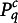 , can control the type-I error under the null hypothesis when *q* is correctly specified (i.e., when 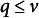 ) and that it increases power as the true treatment effect increases.

We further demonstrate the utility of conditioning on treatment statuses at earlier levels to separate biases among EFs. Our proposed EF analysis is compared to analyses that do not condition on the treatment statuses at earlier levels (i.e., not conditioning on 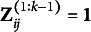 ) while other procedures (e.g., forming strata by matching on observed covariates) remain the same. Each of 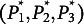 denotes the *p*-value for an unconditioned comparison for each level. For example, 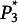 is the *p*-value for an as-treated analysis after controlling for observed covariates through stratification. In the absence of the previous treatment statuses (i.e., at level 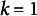 ), 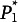 is equivalent to 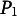 .

In our simulation studies, we consider two designs: one with 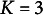 under one-sided non-compliance (Design 1) and the other with 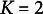 under two-sided non-compliance (Design 2). In each design, we correctly specify *q*, i.e., the number of valid analyses. We also conduct additional simulations with a misspecified *q* to assess its impact on the performance of our approach. See Section S4 of the Supplementary Material for details of the simulation implementation. We present the results for Design 1 (with correctly specified *q*) below, and include additional results with a misspecified *q* in Section S5 of the Supplementary Material. We also include the results for Design 2 in Section S6 of the Supplementary Material. R code for our simulation study is available at: https://github.com/youjin1207/EFinFuzzyRD.

### 6.1 Simulation setup

We generate simulation data for 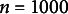 subjects. For each subject, we first generate the baseline covariate and the running variable as follows: 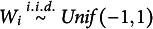 and 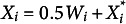 , where 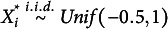 . Then, we generate each subject’s eligibility as 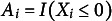 ; that is, each subject *i* is eligible for treatment if and only if 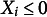 . In addition to the observed covariate, we generate an unmeasured covariate 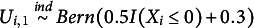 , which may violate the exclusion restriction of the eligibility status ( 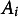 ) on the outcome ( 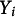 ). Two unmeasured covariates 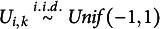 are also generated, each of which can introduce confounding in the treatment assignment at level *k* ( 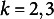 ). Next, we generate the subsequent treatment statuses, 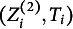 , and the potential outcomes with 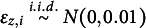 , 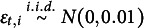 , and 
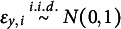
 as follows: 

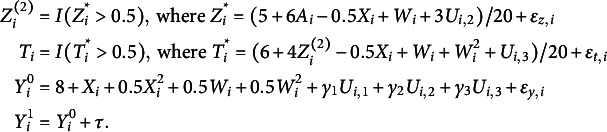

**Figure 4:**
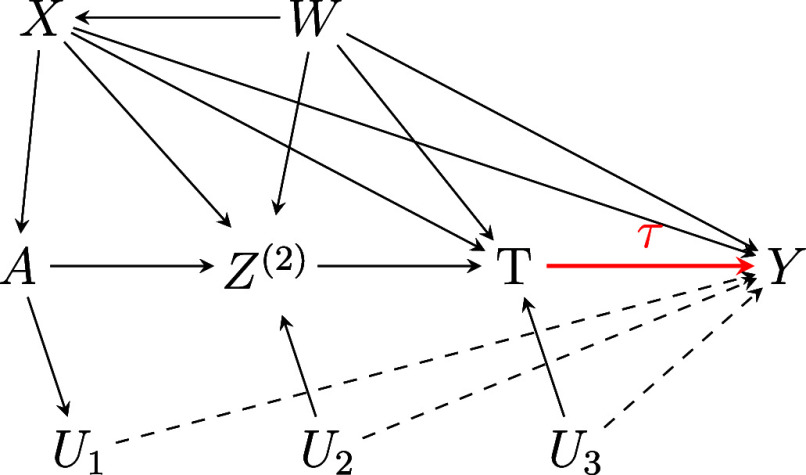
A causal structure of the simulated data. *Note*: The dashed lines indicate the presence of a causal relationship between 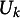 and *Y* when the corresponding 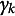 value is non-zero ( 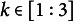 ).

Figure 4 illustrates the causal structure of the observed and unmeasured variables used in our simulation design with 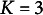 . Our null hypothesis of interest is 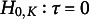 . We conduct one-sided tests, testing the null of no causal effect against the alternative of a greater effect of the treatment. We introduce unmeasured bias through 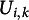 ’s ( 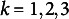 ). For example, the covariate 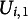 mediates the effect of 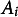 on 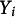 , potentially violating the exclusion restriction by generating a causal pathway between 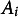 and 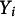 not through 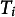 when 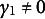 . This bias would not necessarily be avoided by the window selection around the cutoff (i.e., around zero). On the other hand, the covariates 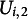 and 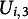 are associated with 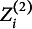 and 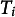 , respectively, potentially confounding their relationships with 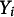 when 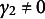 and 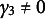 , respectively.^3^

In this simulation design, we consider seven different cases depending on the value of 
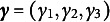
 . In case (1), 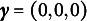 , implying that there is no effect of the three unmeasured covariates on the outcome. In cases (2)–(4), one of the three treatment assignment processes is biased, whereas two of the three are biased in cases (5)–(7). For each case, we report the rejection rates of the three *p*-values from each EF, denoted as 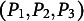 , and their combined *p*-value using Fisher’s method, 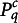 , given a pre-specified *q*. Similarly, we present the results of our comparison analysis, denoted as 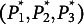 , and their combined *p*-value, 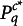 . In case (1), we set 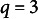 ; in cases (2)–(4), 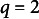 ; and in cases (5)–(7), 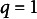 .

### 6.2 Simulation results

**Figure 5:**
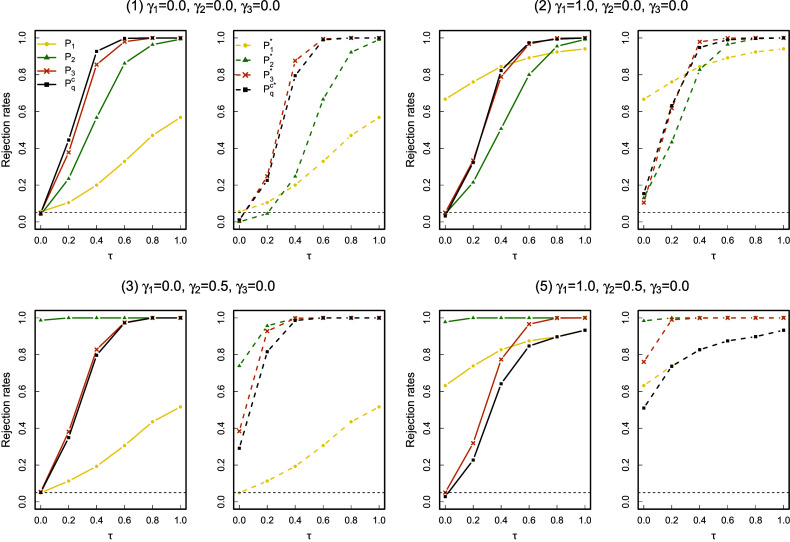
Comparison of rejection rates at the 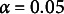 level between the proposed EFs and the unconditioned comparisons, based on 1000 replicates with a sample size of 1000 across four selected cases: (1) 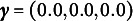 , (2) 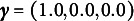 , (3) 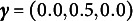 , and (5) 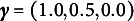 . *Note*: A value of 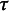 denotes the effect of 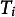 on 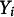 ; 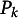 is a *p*-value from each proposed EF for 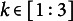 ; 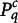 is a combined *p*-value of 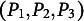 ; 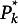 is a *p*-value from each comparison at level *k* without conditioning on 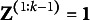 ( 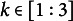 ); 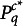 is a combined *p*-value of 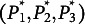 ; 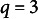 in case (1), 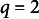 in cases (2) and (3), and 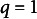 in case (5).

Figure 5 summarizes the results for four out of seven cases: (1) 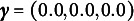 , (2) 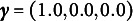 , (3) 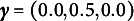 , and (5) 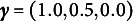 . When all the three treatment assignment processes are unbiased, i.e., in case (1), both the proposed EF analyses and the unconditioned comparisons produce valid *p*-values. We observe that the testing power of the proposed EFs at 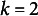 and 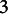 is higher than that of the unconditioned comparisons under our data-generating process. Specifically, in case (1), the unconditioned comparisons of 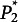 and 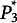 show lower rejection rates than 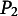 and 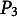 . This might sound counterintuitive, as unconditioned comparisons typically include a larger number of subjects by not conditioning on previous treatment statuses. Our further investigation shows that unconditioned comparisons can produce either conservative or anti-conservative *p*-values depending on simulation scenarios (see Section S5 of the Supplementary Material for additional simulations).

When at least one level of the treatment assignment is biased, the bias from that level does not affect EF analyses at other levels. However, in the unconditioned analyses, bias occurring at level *k* ( 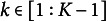 ) may spill over to subsequent levels 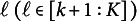 . This expectation is supported by the results in cases (2), (3), and (5). Inflated type-I errors are observed both in the analysis at level *k* where 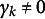 and in subsequent unconditioned comparisons at level 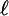 , where 
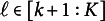
 . Consequently, the combined *p*-value becomes invalid under unconditioned analyses (indicated by dashed black lines) as fewer than *q* unconditioned analyses can be valid. On the other hand, our proposed EFs with a correctly specified number of *q*, i.e., 2 in cases (2) and (3) and 1 in case (5), show that the biased treatment assignment at level *k* does not affect the EF analysis at level 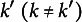 . For example, in case (2), while 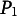 is invalid, 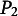 and 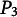 are valid *p*-values. Also, in case (3), while 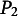 is invalid, 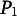 and 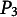 are valid. As a result of such non-overlapping bias across EFs, the combined *p*-values of the EF analyses are still valid in these cases, even without knowing exactly which assignment is biased.

One caveat we note is that the way we generate the assignment variables may lead to misspecification of the assignment models described in Section 4.3, even though they do not necessarily affect the causal assumptions outlined in Section 4.2. Our results demonstrate robustness to such misspecification, although this robustness may not be guaranteed in other settings.

## 7 Empirical examples

In this section, we demonstrate real-world applications of our proposed EF analysis for fuzzy RD designs. The first data analysis evaluates a pre-K program using two different treatment statuses in the context of two-sided non-compliance. The second data analysis evaluates an ETA program using three treatment statuses under a series of one-sided non-compliance.

### 7.1 The effect of New Jersey’s Pre-K program

We use New Jersey’s data from Wong et al. (2007) to evaluate the effect of New Jersey’s Abbott Preschool Program—one of three state-funded pre-K initiatives in New Jersey—on 4-year-old children’s vocabulary skills. In our data analysis, we consider two different treatment statuses: eligibility and use. Eligibility status 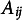 (i.e., 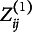 ) represents whether a child is eligible for the pre-K program or not, which is determined by the running variable of a child’s birth date, 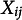 (unit: day); children with birthdays on or after a specific date are eligible for the pre-K program, whereas those with birthdays before it are not. This eligibility status differs from a child’s actual use of or participation in the program, and this non-compliance occurs in a two-side format. Use status 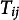 (i.e., 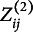 ) represents whether he/she completed the pre-K program in spring 2004. Our outcome of interest is children’s vocabulary scores measured in early fall of the 2004–2005 school year. Measured covariates 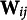 include gender, race/ethnicity, free lunch status, and test language type (English or Spanish). Potential unmeasured covariates ( 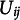 ) include prior abilities and distance to facilities.

In this application, we construct two EFs to test the same null hypothesis ( 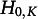 ) that there is no effect from participating in the pre-K program. The first EF compares eligible students ( 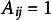 ) and ineligible students ( 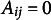 ) in a small neighborhood of the cutoff. This factor is susceptible to bias associated with the eligibility rule under the RD design; for example, ineligible children in New Jersey with high prior abilities might relocate to be eligible for and participate in pre-K programs in other states, thereby “manipulating” the running variable based on unmeasured prior abilities. Moreover, the second EF compares students who participate in the pre-K program ( 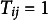 ) and those who do not ( 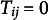 ) among the eligible ( 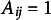 ). This factor is vulnerable to bias associated with children’s actual participation. For example, if the program is located too far away or lacks transportation options, some parents might be unable to assist their children in getting there.

**Figure 6:**
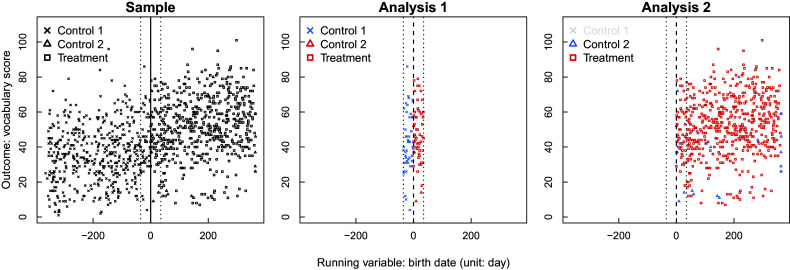
Distributions of three different treatment groups in two EFs analysis in New Jersey’s Pre-K program: for each analysis, red symbols represent the treatment comparison group, while the skyblue symbols represent the control comparison group.

For our data analysis, we first stratify individual children based on the four measured covariates and then conduct a stratified Wilcox rank sum test for each EF. For the first EF, we further constraint our sample near the cutoff by using window selection under the local randomization RD framework. The largest possible window around the cutoff, set at zero, in our data is (−35, 35), which ensures that the *p*-value of the covariate balance test exceeds 0.15. For the first EF, we also use the residual outcome, obtained by regressing the outcome *Y* on *X*, to remove the impact of *X* on *Y* within the window; see Figure 6 for the distributions of three different treatment groups in two EF analyses. Lastly, we combine one-sided *p*-values from multiple EFs using Fisher’s method and conduct sensitivity analyses by varying 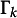 for each factor.

Table 4 presents the results of EF analysis for New Jersey’s pre-K program. In the absence of unmeasured confounding ( 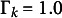 ), the first EF does not reject the null, whereas the second EF rejects the null. The combined *p*-value, calculated using Fisher’s method, is 0.045. Therefore, our EF analysis leads us to reject the null. This suggests a positive effect from participating in the pre-K program. However, sensitivity analyses reveal that adjusting the *p*-values at 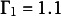 or at 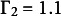 results in combined *p*-values above 0.05. These findings indicate that our causal conclusion are sensitive to even small degrees of unmeasured confounding at 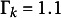 ( 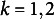 ).

**Table 4:** Results of EFs analysis for New Jersey’s pre-K program

	Evidence factor 1	Evidence factor 2	Fisher’s
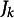 ( 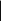 control 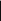 vs. 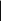 treated 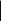 )	8 (51 vs. 57)	9 (21 vs. 555)	
*p*-value ( 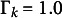 for 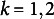 )	0.240	0.032	0.045
*p*-value ( 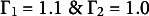 )	0.314	0.032	0.056
*p*-value ( 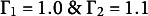 )	0.240	0.048	0.063
*p*-value ( 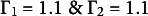 )	0.314	0.048	0.078

*Note*: 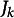 represents the number of strata in EF *k*. 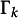 represents the sensitivity parameter in EF *k*. Fisher’s method is used to combine *p*-values from two EFs. 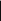 control 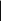 and 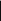 treated 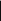 represent the sample size for the control comparison group and treatment comparison group, respectively.

### 7.2 The effect of ETA

We analyze the Grade 8 Mathematics Process Data from the 2017 National Assessment of Educational Progress (NAEP) to study the effect of using ETA on students’ math scores. In this ETA example, we consider three different treatment statuses: eligibility, receipt, and use. Eligibility status 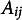 (i.e., 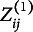 ) is binary and determined by an ordinal running variable. The running variable is ELL English proficiency with six ordinal levels: *No Proficiency, ELL Beginning, ELL Intermediate, ELL Advanced, Formerly ELL,* and *Never ELL*. The eligibility status differs from the receipt status 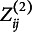 and further differs from the use status 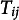 (i.e., 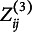 ). We use students’ performance on a 15-item math test block as the outcome 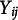 . Measured covariates 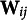 include gender, race/ethnicity, primary language, free lunch status, duration of U.S. school education (less than 1 year or more), and perseverance. Potential unmeasured covariates include students’ years of residence in the US, test anxiety, and academic motivation. In our analysis sample, we exclude students with *Never ELL*, and non-compliance occurs sequentially in a one-sided format.

In this example, we construct three EFs to test the null hypothesis ( 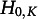 ) that there is no effect of using ETA. The first EF compares eligible students ( 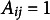 ) and ineligible students ( 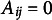 ) in a small neighborhood of the cutoff. This factor is susceptible to bias associated with the eligibility rule under the RD design, as in the pre-K example. The second EF compares students who receive ETA from school administrators ( 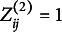 ) and those who do not ( 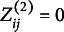 ) among the eligible ( 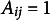 ), and this factor is susceptible to bias associated with school administrator’s decision on ETA receipt. For example, school staff may consider students’ test anxiety levels to determine which students receive ETA in practice. The third EF compares students who actually use ETA ( 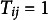 ) versus non-users ( 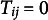 ) among recipients ( 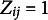 ). This factor is susceptible to bias associated with students’ individual characteristics. For example, students who have higher motivation may actually make use of ETA.

Similar to the pre-K example, we stratify individual students based on the six measured covariates and conduct a stratified Wilcoxon rank sum test for each EF. For the first EF, however, we estimate propensity scores to create a surrogate continuous running variable from the oridinal one, following a recent approach proposed by Li et al. (2021). We select a window of (−0.01, 0.01) around the median of the propensity scores among students with the cutoff category (i.e., *ELL Advanced*). We also use the residual outcome for the first EF. Then, we calculate one-sided *p*-values for each EF and combine them using Fisher’s method. In this application, we use four different treatment groups, similar to those in Figure S2 in Supplementary Appendix S4 from our simulation study, except that we employ the surrogate continuous running variable.

Table 5 provides the results of EFs analysis for ETA. In the absence of unmeasured confounding, all the three EFs do not reject the null, and the combined *p*-value using Fisher’s method is 0.135. As a result, our EF analysis does not reject the null. This concurrence reinforces our conclusion that there is no effect of using ETA on math scores in the NAEP assessment. Since none of the EFs is significant, we do not conduct sensitivity analyses by adjusting 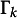 for each factor.

**Table 5:** Results of EF analysis for the ETA

	EF 1	EF 2	EF 3	Fisher’s
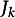 ( 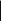 control 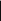 vs. 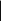 treated 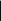 )	6 (40 vs. 80)	10 (510 vs. 120)	10 (90 vs. 30)	
*p*-value	0.231	0.556	0.059	0.135

*Note*: 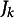 represents the number of strata in EF *k*. Fisher’s method is used to combine *p*-values from two EFs. 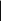 control 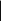 and 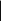 treated 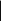 represent the sample size for the control comparison group and treatment comparison group, respectively. Numbers for 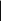 control 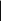 and 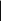 treated 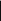 are rounded to nearest tens. Details may not sum to a total due to rounding. *Source*: U.S. Department of Education, National Center on Educational Statistics (NCES), 2017 NAEP Grade 8 Mathematics Process Data, Student Features Data File Partial Form, and Response Data File.

## 8 Discussion and conclusions

In this article, we propose a new EF approach in fuzzy RD designs, which involve sequential treatment assignments and the locality inherent in analyzing samples near the cutoff. This approach is particularly useful when multiple decision-makers are involved in determining treatment statuses sequentially, each producing different types of biases. We establish the causal assumptions required for constructing valid EFs in fuzzy RD designs and demonstrate the effectiveness of our proposed EFs approach through simulation studies by comparing it with unconditional analyses (e.g., as-treated analysis). We also illustrate its effectiveness using two real-world data examples that involve fuzzy RD designs with one-sided and two-sided non-compliance, respectively.

Even though we introduce the setting where the first level of treatment assignment involves RD designs, our proposed approach has broader applicability. More specifically, our approach is applicable to any setting with sequential assignment processes, where one of the comparisons involves an RD analysis. In our ETA example, suppose that ELL English proficiency levels (i.e., running variable) are not available for some students, making it difficult to determine their eligibility status. In this case, the availability for the English proficiency test can be considered as the first treatment status, while the second treatment status is eligibility based on the running variable, which exhibits an RD design. We can still apply our proposed framework in this scenario to construct multiple EFs, as long as the analysis with the RD design properly adjusts the analysis sample to satisfy the local randomization assumption.

There are several limitations in our proposed approach. First, we found that empirical results can be impacted by the way the observations are stratified, which is crucial for randomization-based inference. Therefore, the window selection in the local randomization framework, as well as the choice of matching methods, can impact the empirical results. However, strict criteria for constructing strata, such as using a narrow caliper or imposing a hard threshold for balance tests, may considerably reduce statistical power. Second, we focus on multiple levels of treatment assignment that are potentially affected by individual-level characteristics, e.g., due to individual-level eligibility, constraints, or preferences. However, often such assignment can also be affected by macro-level factors beyond individuals. Future research will formally discuss different sources of biases from multilevel or clustered data in EF analysis, even when treatment is assigned to individual subjects. Third, choosing a presumably valid subset of treatment statuses that satisfy both conditions of Assumption 2 could be challenging in practice. Future research would examine ways to relax this assumption or develop robust methods that are less sensitive to its violation. Lastly, in our simulation and data application studies, we use residuals obtained by regressing the outcome on the running variable (rather than the true errors), which induces correlation between the residuals. As a result, this residual randomization approach remains asymptotically valid under certain conditions (Freedman & Lane, 1983; Sales & Hansen, 2019), even though, in our simulation studies, the rejection rates under the null achieve their nominal levels in finite samples. Despite these limitations, our proposed approach complements the existing RD literature by introducing a novel EF design with sequential treatment assignments embedded in fuzzy RD designs. We hope that our approach serves as a useful tool for reinforcing our causal conclusions in observational studies with fuzzy RD designs.

## Supporting information

Lee and Suk supplementary material Lee and Suk supplementary material

## Data Availability

The datasets used in this article are not publicly available. One dataset is secondary data obtained from Wong et al. (2007), and the other is the restricted-use version of the Grade 8 Mathematics Process Data from the 2017 National Assessment of Educational Progress (NAEP). A restricted-use data license can be obtained through the IES Electronic Application System (https://nces.ed.gov/statprog/instruct.asp).

## Footnotes

1 In the ETA example, compliers mean students who would use (or receive) the ETA program if eligible and would not use (or receive) it if ineligible.

2 In some cases like our pre-K program, treatment assignment  can be determined in the opposite direction such that  if  and  if  .

3 Either the violation of the exclusion restriction or the presence of unmeasured confounders can invalidate an analysis using an IV,  (  ). In practice, we cannot distinguish if an IV analysis is biased due to the violation of the exclusion restriction or to unmeasured confounders.

## Supplementary material

R code for our simulation study and real data applications, along with example datasets, is available at: https://github.com/youjin1207/EFinFuzzyRD.
