# Supplementary material for: Evidence Factors in Fuzzy Regression Discontinuity Designs with Sequential Treatment Assignments
Source: Psychometrika. 2025 Aug 8;90(4):1400–18. doi: 10.1017/psy.2025.10033 (PMC12660022; doi:10.1017/psy.2025.10033)
Supplement: Lee and Suk supplementary material [file S0033312325100331sup001.pdf]

Supplementary Materials

S1 Proof

Proof of Theorem 1. Suppose that each treatment assignment models (2) and (3) in the main text with $\gamma_k = 0$ for $k \in [1:K]$. For each test statistic $t_{\mathcal{D}_k}(\mathbf{Z}^{(k)}, \mathbf{Y})$ for $k \in [1:K]$, the associated p-value P_k is a function of $(\mathbf{Z}^{(1:k)}, \mathbf{Y}, \mathbf{W}, \mathbf{X}) := \mathcal{C}_k$. Under Assumption 1, we have $Pr(P_k \leq p_k) \leq p_k$ with $0 \leq p_k \leq 1$ when the null $H_{0,K}$ is true ($k \in [1:K]$). Moreover, following a structure similar to Lemma 4 of Rosenbaum (2011), we have the following result with $0 \leq p_\ell \leq 1$ for $\ell \in [1:K-1]$:

$$\begin{aligned} Pr(P_{\ell+1} \leq p_{\ell+1} \mid P_{\ell'} \leq p_{\ell'}, \ell' \in [1:\ell]) &= \mathbb{E}\left\{Pr(P_{\ell+1} \leq p_{\ell+1} \mid P_{\ell'} \leq p_{\ell'}, \ell' \in [1:\ell], \mathcal{C}_\ell) | \mathcal{C}_\ell\right\} \\ &= \mathbb{E}\left\{Pr(P_{\ell+1} \leq p_{\ell+1} \mid \mathcal{C}_\ell) \mid P_{\ell'} \leq p_{\ell'}, \ell' \in [1:\ell]\right\} \\ &\leq p_{\ell+1}. \end{aligned}$$

Therefore, the following (in)equalities hold.

$$\begin{aligned} & Pr(P_k \leq p_k; \forall k \in [1:K]) \\ = & \prod_{\ell=1}^{K-1} Pr(P_{\ell+1} \leq p_{\ell+1} \mid P_{\ell'} \leq p_{\ell'}, \ell' \in [1:\ell]) Pr(P_1 \leq p_1) \\ \leq & \prod_{k=1}^{K} p_k. \end{aligned}$$

□

Proof of Theorem 2. For $k \in [1:K]$, since P_k is a function of $\mathbf{Z}_{ij}^{(1:k-1)}$ with $Z_{ij}^{(1:0)} \equiv \emptyset$, invalidity in $\mathbf{Z}_{ij}^{(1:k-1)}$ does not affect the validity of a comparison with $Z_{ij}^{(k)}$. Moreover, for $k \in [1:K-1]$, the invalidity of $Z_{ij}^{(k+1:K)}$ does not affect the validity of $Z_{ij}^{(k)}$ by Assumption 2. Thus, $Pr(P_k \leq p_k) \leq p_k$ for $k \in \mathcal{V}$ regardless of $\mathcal{V}$. Then applying Theorem 1 to $\{P_k : k \in \mathcal{V}\}$, $Pr(P_k \leq p_k; k \in \mathcal{V}) \leq \prod_{k \in \mathcal{V}} p_k$. □

S2 Relaxing Condition (b) of Assumption 2

When the presence of unmeasured common causes is suspected among K treatment statuses that satisfying Assumption 2(a), one can choose a subset of treatment statuses within which Assumption 2(b) holds. Depending on the assumed common causes among the K statuses, multiple subsets may satisfy Assumption 2(b).

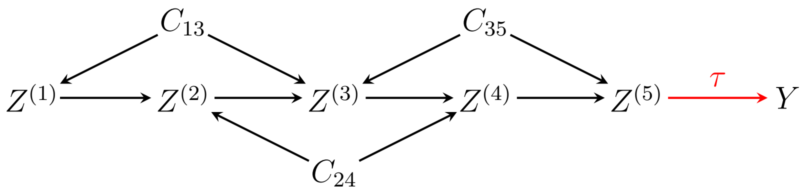

Figure S1. A directed acyclic graph (DAG) illustrating the causal relationships among treatment statuses $Z^{(k)}$ ($k \in [1 : K]$) with $K = 3$, outcome Y, and known common causes C_{13}, C_{24}, and C_{35}. Observed covariates are omitted for simplicity.

Consider the case illustrated in Figure S1 with K=5 statuses and the three presumed unmeasured common causes, C_{13}, C_{24}, and C_{35}. In this case, one can choose $(Z^{(1)}, Z^{(2)}, Z^{(5)})$, $(Z^{(1)}, Z^{(4)}, Z^{(5)})$, or even $(Z^{(3)}, Z^{(4)})$ as a set of treatment statuses to construct evidence factors. When deciding which statuses to use, several factors can be considered before analysis, such as sample sizes (e.g., the proportion between treatment and control comparison groups), plausibility of Assumption 1, and the strength as an IV for the treatment effect of interest. For example, one may prefer using $(Z^{(1)}, Z^{(4)}, Z^{(5)})$ over the other two suggested sets, as this set would provide three evidence factors. It includes $Z^{(5)}$, which does not require the exclusion restriction-type assumption (i.e., Assumption 1), and $Z^{(4)}$, which is expected to act as a stronger IV for $Z^{(5)}$ than $Z^{(2)}$. When $Z^{(k)}$ is used to construct an evidence factor, the analysis must condition on $\mathbf{Z}^{(1:k-1)} = 1$, even if not all statuses in $\mathbf{Z}^{(1:k-1)}$ are not used for evidence factors analysis ($k \in [1 : K]$).

S3 Remarks on treatment assignment models

In the main text, we consider the following assignment models at level $k = 1$:

$$Pr(A_{ij} = 1 \mid \mathcal{F}, \mathcal{W}) = \frac{\exp\{\kappa_1(\mathbf{w}_{ij}) + \gamma_1 u_{ij,1}\}}{1 + \exp\{\kappa_1(\mathbf{w}_{ij}) + \gamma_1 u_{ij,1}\}}, \tag{S1}$$

and, and for subsequent levels of $k \in [2 : K]$, we consider:

$$Pr(Z_{ij}^{(k)} = 1 \mid \mathcal{F}, \mathbf{Z}_{ij}^{(1:k-1)} = \mathbf{1}) = \frac{\exp\{\kappa_k(\mathbf{w}_{ij}) + \gamma_k u_{ij,k}\}}{1 + \exp\{\kappa_k(\mathbf{w}_{ij}) + \gamma_k u_{ij,k}\}}. \tag{S2}$$

Compared to the model (S1), the assignment model (S2) does not utilize a running variable for assignment, so it does not require conditioning on the window around the cutoff. However, if it is believed that the assignment mechanisms at subsequent levels might vary substantially based on their running variable values, then the following model could be considered at the cost of sample size, the extent to which depends on the width of the window.

$$Pr(Z_{ij}^{(k)} = 1 \mid \mathcal{F}, \mathbf{Z}_{ij}^{(1:k-1)} = \mathbf{1}, \mathcal{W}) = \frac{\exp\{\kappa_k^*(\mathbf{w}_{ij}) + \gamma_k^* u_{ij,k}\}}{1 + \exp\{\kappa_k^*(\mathbf{w}_{ij}) + \gamma_k^* u_{ij,k}\}}. \tag{S3}$$

A value of two parameters, κ_k in (S2) and κ_k^* in (S3), can be different when subjects within the window having the same value of $\mathbf{w}_{ij}$ and $u_{ij,k}$ do not necessarily have the same value of a running variable x_{ij} and/or when x_{ij} affects the treatment assignment at level k. Alternatively, one can consider using a running variable X_{ij} as a matching covariate to construct strata, assuming the following assignment model.

$$Pr(Z_{ij}^{(k)} = 1 \mid \mathcal{F}, X_{ij}, \mathbf{Z}_{ij}^{(1:k-1)} = \mathbf{1}) = \frac{\exp\{\kappa_k^\dagger(\mathbf{w}_{ij}, x_{ij}) + \gamma_k^\dagger u_{ij,k}\}}{1 + \exp\{\kappa_k^\dagger(\mathbf{w}_{ij}, x_{ij}) + \gamma_k^\dagger u_{ij,k}\}}. \tag{S4}$$

This assignment model views the running variable as a potential confounder but permits matching subjects with slightly different values in X_{ij} outside of the window $\mathcal{W}$ unless

exact matching is used on the running variable.

S4 Simulation implementations

In this section, we demonstrate how we construct three evidence factors to obtain one-sided p-values, (P_1, P_2, P_3), testing the same null hypothesis using a single dataset in our current simulation design. First, we construct strata based on similarities in Mahalanobis distance in X_i and W_i using `R` package `quickmatch`, which allows full matching with multiple treatment groups (Savje et al., 2017). We restrict our study sample to have at most one Mahalanobis distance within strata (i.e., caliper = 1).

Figure S2 visualizes the distribution and the application of four different treatment groups in the three evidence factors analyses within a simulated dataset of $n = 1000$ subjects. This figure illustrates how multiple evidence factors can leverage the study observations in each analysis. The first analysis, shown in Figure S2b, compares the control and treatment comparison groups (denoted by skyblue and red colors, respectively) within the window specified by the two vertical dotted lines around the cutoff of zero. Under the the local randomization framework, subjects within the window are assumed to be randomly assigned to either group. However, even within the window, there could still be imbalances in each of the observed covariates in practice with a finite sample. By comparing subjects within strata constructed through the observed covariates, we further adjust for these covariates within the window. For Analysis 1, we use the residuals from the linear regression of Y_{ij} on X_{ij} as transformed outcomes to eliminate the remaining dependency between the observed outcome and the running variable (Sales & Hansen, 2019).

As shown in Figures S2c and S2d, the evidence factors analyses at levels $k = 2$ and 3 can rely on a relatively small number of samples, even without the window restriction, if there are a substantial number of ineligible subjects (Control 1) in data. Although the total sample size used in Analysis 2 is larger than that in Analysis 3 by including subjects

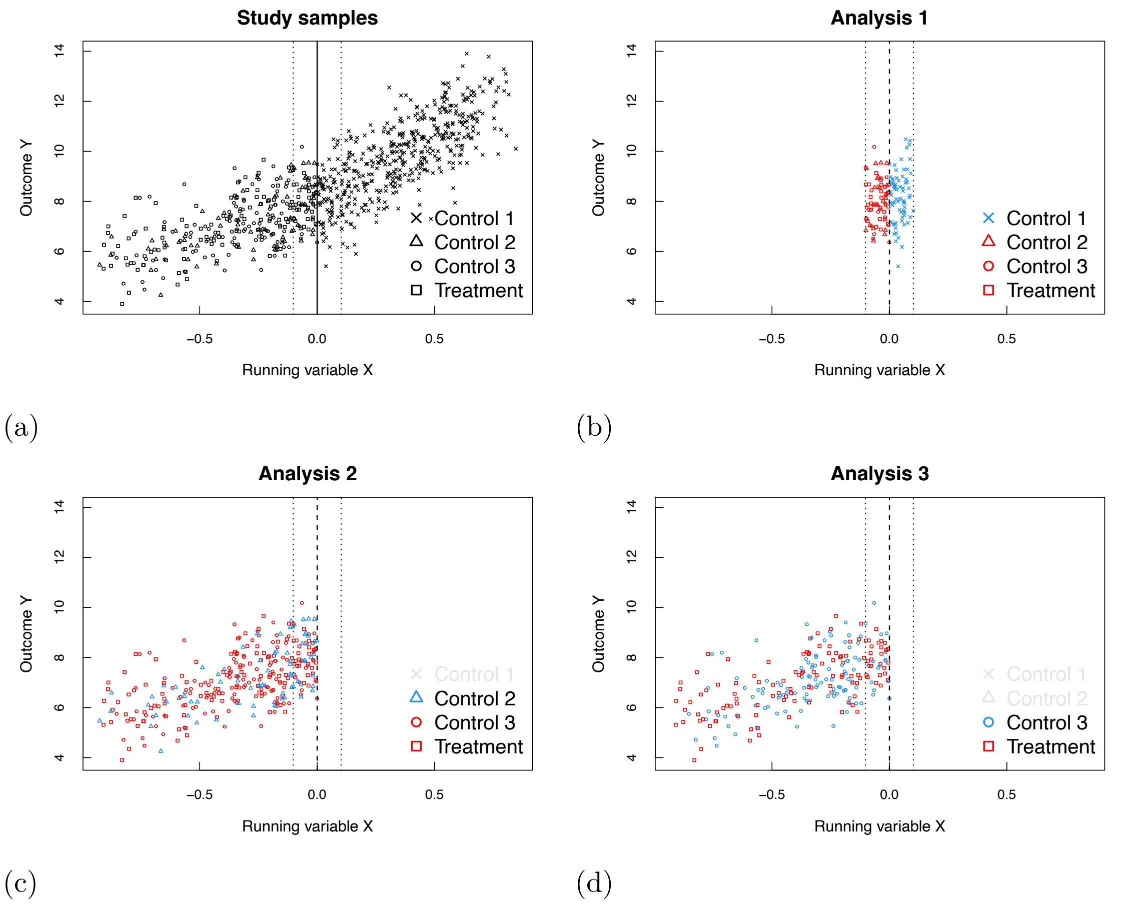

Figure S2. The use of four different treatment groups in the three evidence factor analyses: for each analysis, red symbols represent the treatment comparison group, while the skyblue symbols represent the control comparison group.

from Control 2, Analysis 2 often has a reduced power compared to Analysis 3 for two reasons. First, the sample size of the control comparison group (depicted as skyblue triangles in Figure S2c) in Analysis 2 could be small. Second, the treatment comparison group includes subjects in Control 3 as well as those treated. Thus, the treatment effect detected from Analysis 2 is easily diluted compared to the direct comparison in Analysis 3, where the treated subjects are compared to Control 3 only. `R` codes for our simulation study can be found at the first author's GitHub repository.

We implement the proposed evidence factors analysis to obtain (P_1, P_2, P_3) and their combined p-value, P_q^c, using Fisher's method. For comparison, we also conduct the unconditional procedure to obtain (P_1^*, P_2^*, P_3^*) and their combined p-value, P_q^{c*}. This

procedure is replicated 1000 independent times for seven different cases, and rejection rates of the null hypothesis (no treatment effect) are then calculated.

S5 Additional simulation results under one-sided non-compliance

Our proposed evidence factors analysis is compared to analyses that do not condition on the treatment statuses at earlier levels (i.e., not conditioning on $\mathbf{Z}_{ij}^{(1:k-1)} = \mathbf{1}$). Supplementary Table S1 illustrates the use of four groups in these unconditioned comparisons. Unlike the evidence factors analysis (as shown in Tables 2 and 3 in the main text), the control comparison group at level k ($k \in [2:K]$) now includes those who did not take a treatment at previous levels, indicated by blue text in Table S1. Without previous levels to condition on, the first analysis that utilizes the earliest treatment assignment remains the same as in the evidence factors analysis.

Table S1

The treatment and control comparison groups used for each unconditioned comparison (UC)

$X_{ij} \in \mathcal{W}$	$Z_{ij}^{(1)}$	$Z_{ij}^{(2)}$	$Z_{ij}^{(3)}$	Group	UC 1	UC 2	UC 3
1	0	0	0	Control 1	C	C	C
1	1	0	0	Control 2	T	C	C
1	1	1	0	Control 3	T	T	C
1	1	1	1	Treatment	T	T	T
0	0	0	0	Control 1	·	C	C
0	1	0	0	Control 2	·	C	C
0	1	1	0	Control 3	·	T	C
0	1	1	1	Treatment	·	T	T

NOTE: T: Treatment comparison group; C: Control comparison group; ·: Data excluded; blue colored strata are added in the unconditioned comparison compared to the evidence factors analysis

Tables S2 and S3 present the rejection rates across different values of the true treatment effect τ for each case using the proposed evidence factors analysis and the unconditioned comparisons, respectively, across seven cases considered in the main text. In case (1), where there is no unmeasured confounding in all three treatment assignment processes, all three p-values, as well as their combined p-value, effectively control the type-I

error at $\alpha = 0.05$ under the null (i.e., $\tau = 0.0$) using either approaches. As expected, the evidence factor at $k = 3$, which directly compares the treated and control subjects, achieves the highest testing power, while the evidence factor at $k = 1$ of the RD analysis generally has the lowest power. The unconditioned analyses at levels $k = 2$ and 3 exhibit small power than those in evidence factors analysis.

Table S2

Rejection rates at $\alpha = 0.05$ level of the proposed evidence factors approach, based on 1000 replicates with $n = 1000$

$\tau = 0.0$							
$\boldsymbol{\gamma} = (\gamma_1, \gamma_2, \gamma_3)$	(1) (0,0,0)	(2) (1.0, 0, 0)	(3) (0, 0.5, 0)	(4) (0, 0, 0.5)	(5) (1.0, 0.5, 0)	(6) (1.0, 0, 0.5)	(7) (0, 0.5, 0.5)
With P_1	0.055	0.667	0.049	0.054	0.632	0.647	0.055
P_2	0.039	0.043	0.987	0.041	0.978	0.041	0.984
P_3	0.049	0.050	0.054	0.913	0.050	0.879	0.903
With P_q^c	0.047	0.032	0.051	0.041	0.028	0.025	0.053
$\tau = 0.2$							
With P_1	0.105	0.761	0.113	0.103	0.739	0.731	0.107
P_2	0.235	0.215	1.000	0.219	1.000	0.206	1.000
P_3	0.378	0.334	0.381	0.995	0.319	0.985	0.993
With P_q^c	0.445	0.323	0.349	0.213	0.227	0.156	0.104
$\tau = 0.4$							
With P_1	0.200	0.845	0.193	0.182	0.827	0.827	0.164
P_2	0.567	0.507	1.000	0.515	1.000	0.459	1.000
P_3	0.855	0.789	0.828	1.000	0.774	1.000	1.000
With P_q^c	0.927	0.823	0.797	0.537	0.642	0.334	0.164
$\tau = 0.6$							
With P_1	0.329	0.892	0.306	0.308	0.875	0.865	0.282
P_2	0.862	0.800	1.000	0.798	1.000	0.732	1.000
P_3	0.980	0.967	0.975	1.000	0.966	1.000	1.000
With P_q^c	0.998	0.974	0.973	0.824	0.847	0.637	0.282
$\tau = 0.8$							
With P_1	0.470	0.924	0.435	0.429	0.898	0.900	0.382
P_2	0.964	0.955	1.000	0.942	1.000	0.922	1.000
P_3	1.000	0.999	1.000	1.000	0.999	1.000	1.000
With P_q^c	1.000	0.994	1.000	0.965	0.897	0.829	0.382

NOTE: A value of τ denotes the effect of T_i on Y_i; seven cases are considered depending on a value of $\boldsymbol{\gamma} = (\gamma_1, \gamma_2, \gamma_3)$, where γ_k denotes the impact of unmeasured $U_{i,k}$ on Y_i for $k \in [1:3]$; P_k is a one-sided p-value from each proposed evidence factor for $k \in [1:3]$; P_q^c is a combined p-value of (P_1, P_2, P_3) given a pre-specified q; $q = 3$ in case (1), $q = 2$ in cases (2)-(4), and $q = 1$ in cases (5)-(7).

When only one treatment assignment process is biased (i.e., cases (2)-(4)) in Table S2, the corresponding evidence factor at level k with a non-zero γ_k exhibits an inflated type-I error under the null, while others remain unaffected. With q correctly specified to 2, the combined p-value of the evidence factors analysis is still valid, and its power is generally as high as the highest among the two valid p-values. This is possible due

Table S3

Rejection rates at $\alpha = 0.05$ level of the unconditioned comparisons, based on 1000 replicates with $n = 1000$

$\tau = 0.0$							
$\boldsymbol{\gamma} = (\gamma_1, \gamma_2, \gamma_3)$	(1) (0,0,0)	(2) (1.0, 0, 0)	(3) (0, 0.5, 0)	(4) (0, 0, 0.5)	(5) (1.0, 0.5, 0)	(6) (1.0, 0, 0.5)	(7) (0, 0.5, 0.5)
With P_1^*	0.055	0.667	0.049	0.054	0.632	0.647	0.055
P_2^*	0.000	0.130	0.739	0.000	0.984	0.125	0.984
P_3^*	0.007	0.105	0.384	0.423	0.761	0.800	0.962
With P_q^{c*}	0.011	0.154	0.291	0.006	0.510	0.105	0.053
$\tau = 0.2$							
With P_1^*	0.105	0.761	0.113	0.103	0.739	0.731	0.107
P_2^*	0.045	0.433	0.957	0.045	1.000	0.416	0.946
P_3^*	0.248	0.618	0.928	0.943	0.990	0.993	1.000
With P_q^{c*}	0.226	0.632	0.816	0.101	0.737	0.335	0.107
$\tau = 0.4$							
With P_1^*	0.200	0.845	0.193	0.182	0.827	0.827	0.164
P_2^*	0.247	0.828	0.998	0.224	1.000	0.787	0.996
P_3^*	0.876	0.979	0.998	1.000	0.999	1.000	1.000
With P_q^{c*}	0.795	0.948	0.986	0.308	0.827	0.668	0.164
$\tau = 0.6$							
With P_1^*	0.329	0.892	0.306	0.308	0.875	0.865	0.282
P_2^*	0.666	0.965	1.000	0.615	1.000	0.944	1.000
P_3^*	0.995	0.999	1.000	1.000	1.000	1.000	1.000
With P_q^{c*}	0.990	0.990	1.000	0.657	0.875	0.828	0.282
$\tau = 0.8$							
With P_1^*	0.470	0.924	0.435	0.429	0.898	0.900	0.382
P_2^*	0.922	0.996	1.000	0.892	1.000	0.991	1.000
P_3^*	1.000	1.000	1.000	1.000	1.000	1.000	1.000
With P_q^{c*}	1.000	0.998	1.000	0.900	0.898	0.893	0.382

NOTE: A value of τ denotes the effect of T_i on Y_i; seven cases are considered depending on a value of $\boldsymbol{\gamma} = (\gamma_1, \gamma_2, \gamma_3)$, where γ_k denotes the impact of unmeasured $U_{i,k}$ on Y_i for $k \in [1:3]$; P_k^* is a one-sided p-value from each comparison at level k without conditioning on $Z^{(k-1)}$ ($k \in [1:3]$); P_q^{c*} is a combined p-value of (P_1^*, P_2^*, P_3^*) given a pre-specified q; $q = 3$ in case (1), $q = 2$ in cases (2)-(4), and $q = 1$ in cases (5)-(7).

to the near independence property, even if we do not know which of the three evidence factors is invalid. On the other hand, cases (2)-(4) in Table S3 show that a bias in the treatment assignment at level k ($k \in [1 : K-1]$) may spill over into the unconditioned comparisons at subsequent levels ℓ ($\ell \in [k+1 : K]$). For instance, in case (3), where the treatment assignment at level $k = 2$ is biased, this does not affect the analysis at level $k = 1$ but invalidates the unconditioned analysis at level $k = 3$, inflating the type-I error under the null. Therefore, their combined p-value at q (e.g., $q = 2$ under cases (2)-(4)) may be invalid, as the number of valid unconditioned analyses could be less than q. In case (4), a biased treatment assignment process at level $k = 3$ would not affect the analyses at $k = 1$

and $k = 2$, resulting in a valid combined p-value P_q^{c*} with $q = 2$.

Similarly, in cases (5)-(7), where two out of three treatment assignments are biased, Table S2 shows that at least one evidence factor remains valid, resulting in a valid combined p-value with $q = 1$ using evidence factors analysis. The testing power in this case is significantly lower than in cases with $q = 3$ or 2, as it relies on the largest (i.e., most conservative) p-value among the three. However, it effectively controls the type-I error under the null, even without knowing which evidence factors are biased. On the other hand, the results in Table S3 show that unless we know which treatment assignments are biased, we may obtain invalid three p-values under the unconditioned comparisons due to not properly conditioning on the source of bias from the previous assignment processes.

Moreover, the results in Table S3 demonstrate the conservativeness of the unconditioned comparisons of P_2^* and P_3^*, showing lower rejection rates than P_2 and P_3. This might sound counterintuitive, as unconditioned comparisons typically include a larger number of subjects by not conditioning on previous treatment statuses. In fact, unconditioned comparisons may result in either conservative or anti-conservative p-values due to the inclusion of ineligible subjects as controls at level k ($\in [2 : K]$). Ineligible subjects (i.e., Control 1 in Table 3 in the main text) have a very different distribution of the running variable compared to other subjects, violating the positivity assumption commonly assumed in causal inference. Therefore, even with sophisticated matching methods, the values of the running variable for ineligible subjects cannot be identical or similar to those of other subjects within the same strata. This violation of covariate balance does not affect the RD analysis much as it restricts the range of the running variable for both comparison groups. However, it may impact other analyses if ineligible subjects are used for comparison.

Table S4 presents the combined results for a pre-specified $q \in [1 : 3]$ under the null hypothesis with $\tau = 0.0$. When q is over-specified (e.g., $q < 3$ in case (1)), the combined p-values remain valid but are overly conservative. On the other hand, if q is less than the

Table S4

Rejection rates at $\alpha = 0.05$ level of the proposed evidence factors approach under the null, based on 1000 replicates with $n = 1000$ across q values

$\tau = 0.0$							
$\boldsymbol{\gamma} = (\gamma_1, \gamma_2, \gamma_3)$	(1) (0,0,0)	(2) (1.0, 0, 0)	(3) (0, 0.5, 0)	(4) (0, 0, 0.5)	(5) (1.0, 0.5, 0)	(6) (1.0, 0, 0.5)	(7) (0, 0.5, 0.5)
With P_1	0.055	0.667	0.049	0.054	0.632	0.647	0.055
P_2	0.039	0.043	0.987	0.041	0.978	0.041	0.984
P_3	0.049	0.050	0.054	0.913	0.050	0.879	0.903
With $P^c_{q=1}$	0.000	0.002	0.002	0.005	0.027	0.032	0.053
With $P^c_{q=2}$	0.004	0.028	0.053	0.052	0.491	0.376	0.772
With $P^c_{q=3}$	0.047	0.451	0.950	0.780	0.981	0.917	0.997

NOTE: A value of τ denotes the effect of T_i on Y_i; seven cases are considered depending on a value of $\boldsymbol{\gamma} = (\gamma_1, \gamma_2, \gamma_3)$, where γ_k denotes the impact of unmeasured $U_{i,k}$ on Y_i for $k \in [1:3]$; P_k is a one-sided p-value from each proposed evidence factor for $k \in [1:3]$; P^c_q is a combined p-value of (P_1, P_2, P_3) given a pre-specified q.

actual number of valid evidence factors (e.g., $q = 3$ in cases (2)-(4) and $q > 2$ in cases (5)-(7)), the combined p-values fail to control the type-I error under the null hypothesis.

We further investigate the impact of including ineligible subjects in the unconditioned comparisons. We hypothesize that unconditioned comparisons may yield either conservative or anti-conservative p-values, depending on how the running variable affects the outcome. This is due to the uncontrolled imbalance in the running variable between ineligible and eligible subjects, even after matching. We particularly investigate how the relationship between the running variable X and the outcome Y impacts the evidence factors analysis and unconditioned comparisons. We vary the parameter value of κ_1 in the following outcome generating model. We set $\kappa_2 = 0.5\kappa_1$.

$$
\begin{aligned}
Y_i^0 &= 8 + \kappa_1 X_i + \kappa_2 X_i^2 + 0.5W_i + 0.5W_i^2 + \gamma_1 U_{i,1} + \gamma_2 U_{i,2} + \gamma_3 U_{i,3} + \epsilon_{y,i} \\
Y_i^1 &= Y_i^0 + \tau.
\end{aligned}
$$

Figure S3 shows the rejection rates of the proposed evidence factors analysis and the unconditioned comparisons under the null across different values of κ_1. In our data generating process presented in the main text, where $\kappa_1 = 1.0$, a running variable X has a positive effect on the outcome; the unconditioned analyses at k ($k \in [2 : K]$) include

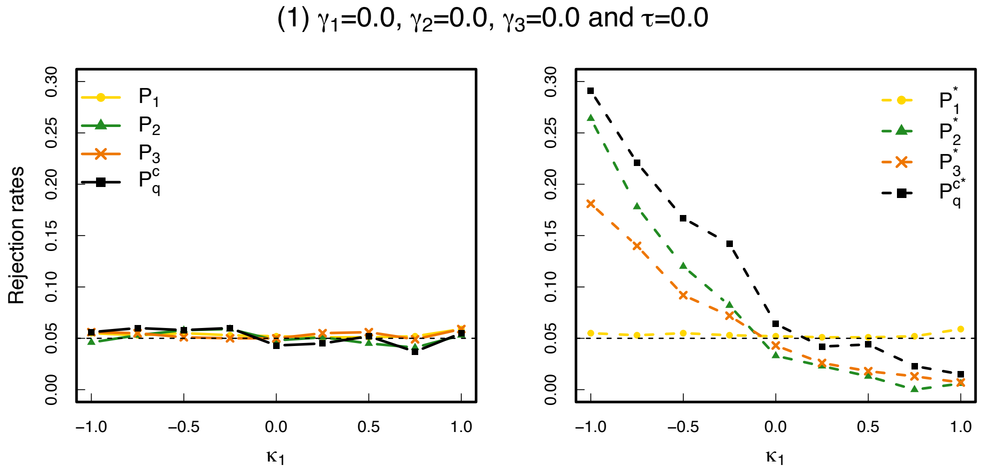

Figure S3. Rejection rates under the null when varying the parameter value of κ_1 from -1.0 to 1.0. We set $\kappa_2 = 0.5\kappa_1$. P_k is a p-value from each proposed evidence factor for $k \in [1:3]$; P_q^c is a combined p-value of (P_1, P_2, P_3); P_k^* is a p-value from each comparison at level k without conditioning on $\mathbf{Z}^{(1:k-1)} = \mathbf{1}$ $(k \in [1:3])$; P_q^{c*} is a combined p-value of (P_1^*, P_2^*, P_3^*); $q = 3$ in case (1) where no treatment assignment is biased.

ineligible subjects with positive values of X in the control group. Consequently, including these controls in the analysis would enhance the positive effect among the controls, providing more favorable evidence of the null hypothesis of no treatment effect when implementing an one-sided test for a greater effect of the treatment. The results of P_2^* and P_3^* in Figure S3, therefore, are conservative showing far less type-I error than $\alpha = 0.05$ level.

On the other hand, when the running variable X has a negative effect on the outcome (i.e., a negative value of κ_1), ineligible subjects with positive values of X tend to have lower outcome values. In this scenario, including these subjects in the control comparison groups strengthens the evidence against the null hypothesis, indicating a greater treatment effect. For this reason, the results of P_2^* and P_3^* in Figure S3 demonstrate inflated type-I errors. However, the corresponding evidence factors analyses (i.e., the results of P_2 and P_3) remain valid regardless of the value of κ_1. This is because evidence factors analyses at k $(k \in [2:K])$ do not include ineligible subjects in their comparisons.

S6 Simulation under two-sided non-compliance

In this section, we consider two-sided non-compliance with $K = 2$ and $n = 1000$ subjects. We generate the baseline covariate and the running variable similarly to the one-sided non-compliance case: $W_i \overset{i.i.d.}{\sim} Unif(-1, 1)$ and $X_i = 0.5W_i + X_i^*$, where $X_i^* \overset{i.i.d.}{\sim} Unif(-0.5, 1)$. We consider the case where each subject i is eligible for treatment if and only if $X_i \leq 0$, i.e., $A_i = I(X_i \leq 0)$. With $K = 2$, we generate two unmeasured covariates that can confound each treatment assignment process, $U_{i,1}$ and $U_{i,2}$, where $U_{i,1} \overset{ind}{\sim} Bern(0.5I(X_i \leq 0) + 0.3)$ and $U_{i,2} \overset{i.i.d.}{\sim} Unif(-1, 1)$. We generate the treatment status T_i as $I(T_i^* > 0.5)$, where

$$T_i^* = \begin{cases} \text{logit}^{-1}\left(-2 + 3A_i + 0.5X_i - 0.5W_i + 3U_{i,2} + e_i\right) & \text{when } A_i = 1 \\ \text{logit}^{-1}\left(-2 + 3A_i + 0.5X_i - 0.5W_i + 2U_{i,2} + U_i^* + e_i\right) & \text{when } A_i = 0 \end{cases}$$

with $\text{logit}^{-1}(x) = \{1 + \exp(-x)\}^{-1}$, $U_i^* \overset{i.i.d.}{\sim} Unif(-1, 1)$ and $e_i \overset{i.i.d.}{\sim} N(0, 1)$. The potential outcomes are generated as follows with $\epsilon_{y,i} \overset{i.i.d.}{\sim} N(0, 1)$:

$$\begin{aligned} Y_i^0 &= 8 - 0.5X_i + 0.5X_i^2 + 0.5W_i + 0.5W_i^2 + \gamma_1 U_{i,1} + \gamma_2 U_{i,2} + U_i^* + \epsilon_{y,i} \\ Y_i^1 &= Y_i^0 + \tau. \end{aligned}$$

Compared to the setting considered in Section 6 in the main text, having $T_i = 1$ does not necessarily indicate $A_i = 1$. An additional (presumably unmeasured) covariate U_i^* confounds the effect of T_i and Y_i among $A_i = 0$. This represents one of the unmeasured factors that impacts the treatment use status among ineligible subjects with $A_i = 0$; the other unmeasured factor is $U_{i,2}$ when $\gamma_2 \neq 0$, which also could confound the treatment assignment process among eligible subjects with $A_i = 1$. Since our second proposed evidence factor with T_i relies only on the treatment assignment among the eligible, the presence of the unmeasured confounder U_i^* should not affect our proposed methods. On the other hand, when $\gamma_1 = \gamma_2 = 0$, there is no unmeasured confounding induced by $U_{i,k}$

($k = 1, 2$) in the treatment assignments we leverage. However, when $\gamma_k \neq 0$, there could be bias in the evidence factor at level k ($k = 1, 2$).

In this simulation design, we examine the type-I error and testing power under three different cases: (i) $\gamma_1 = \gamma_2 = 0.0$; (ii) $\gamma_1 = 0.5$ and $\gamma_2 = 0.0$; and (iii) $\gamma_1 = 0.0$ and $\gamma_2 = 0.5$. For each case, we calculate the rejection rates based on 1000 replicates using the p-values of two evidence factors: (a) P_1 obtained from the evidence factor at $k = 1$; (b) P_2 obtained from the evidence factor at $k = 2$; and (c) the combined p-value P_q^c of P_1 and P_2, assuming at least q valid evidence factors. Under case (i) we set $q = 2$, and under cases (ii)-(iii) we set $q = 1$. We also compare these results of the proposed evidence factors design with a direct comparison that uses T_i only. The fourth p-value of, (d) P_2^*, compares those who use the treatment and those who do not, regardless of their eligibility status, after stratification on the observed covariates.

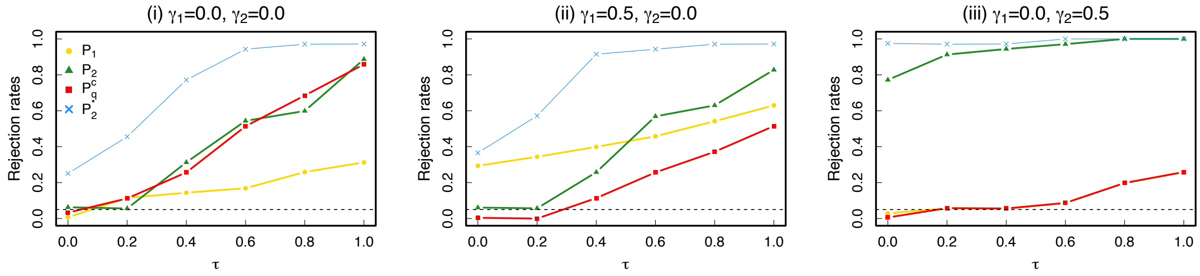

Figure S4. Rejection rates of four different methods across three different scenarios

Figure S4 presents the rejection rates across different values of the true causal effect τ for each case. Under case (i), where there is no unmeasured confounding in either treatment assignment process, the results from each evidence factor (i.e., those based on P_1 and P_2) successfully control the type-I error at $\alpha = 0.05$, so does the combined p-value, P_q^c at $q = 2$. The combined p-value is as powerful as the most powerful evidence factor, which in this case is the factor at $k = 2$. Under case (ii), where the treatment assignment process at level $k = 1$ is biased, the rejection rate based on P_1 exhibits an inflated type-I error under the null, while each of the second evidence factor and the combined one assuming $q = 1$ provides valid p-values. We observe similar results under case (iii), where the

treatment assignment process at level $k = 2$ is biased. The validity of the first evidence factor and the combined one is not affected by the unmeasured confounding in the treatment assignment at level $k = 2$.

Across the three cases, a simple comparison using T_i that results in a p-value of P_2^* leads to an inflated type-I error under the null (i.e., when $\tau = 0.0$). This is because, regardless of a value of γ_2, there exists an unmeasured confounder U_i^* that only affects ineligible subjects. Therefore, if it is suspicious that unobserved factors could influence treatment use status among ineligible subjects, an as-treated analysis is easily biased. On the other hand, if researchers have knowledge about factors that affect the treatment use status among the eligible, two evidence factors can be constructed and those can produce a valid p-value, even if one of the two treatment assignment processes is biased.

References

Rosenbaum, P. R. (2011). Some approximate evidence factors in observational studies. *Journal of the American Statistical Association*, *106*(493), 285–295. https://doi.org/10.1198/jasa.2011.tm10422

Sales, A. C., & Hansen, B. B. (2019). Limitless regression discontinuity. *Journal of Educational and Behavioral Statistics*, *45*(2), 143–174. https://doi.org/10.3102/1076998619884904

Savje, F., Sekhon, J., & Higgins, M. (2017, May). *Quickmatch: Quick generalized full matching*. The R Foundation. https://doi.org/10.32614/cran.package.quickmatch
